# Overall survival in Japanese patients with ER+/HER2− advanced breast cancer treated with first-line palbociclib plus letrozole

**DOI:** 10.1007/s12282-023-01511-z

**Published:** 2023-10-26

**Authors:** Masato Takahashi, Tomofumi Osako, Hiroyuki Yasojima, Kenichi Inoue, Masahiro Kawashima, Hideki Maeda, Akemi Ichikawa, Yasuaki Muramatsu, Norikazu Masuda

**Affiliations:** 1 Breast Surgery, Hokkaido University Hospital, Sapporo, Japan; 2 Breast Center, Kumamoto Shinto General Hospital, Kumamoto, Japan; 3 Department of Surgery, Breast Oncology, National Hospital Organization Osaka National Hospital, Osaka, Japan; 4 Division of Breast Oncology, Saitama Cancer Center, Saitama, Japan; 5 Department of Breast Surgery, Graduate School of Medicine, Kyoto University, Kyoto, Japan; 6 Department of Breast Surgery, National Hospital Organization Hokkaido Cancer Center, Sapporo, Japan; 7 Medical Affairs, Pfizer Japan Inc., Tokyo, Japan; 8 Department of Breast and Endocrine Surgery, Graduate School of Medicine, Nagoya University, Nagoya, Japan

**Keywords:** Palbociclib, Breast cancer, ER+/HER2−, Overall survival, Japan

## Abstract

**Background:**

An open-label, single-arm, Japanese phase 2 study (J-Ph2) investigated the efficacy and safety of first-line (1L) palbociclib (PAL) + letrozole (LET) in postmenopausal Japanese women with ER+/HER2− advanced breast cancer (ABC). In the final analysis, median progression-free survival was 35.7 months (95% CI 21.7–46.7); but overall survival (OS) data were immature. Here, we report the findings from a follow-up study of J-Ph2 (NCT04735367) evaluating OS and subsequent therapy in these Japanese women.

**Methods:**

Patients (N = 42) who participated in J-Ph2 were enrolled in the OS follow-up study. The primary endpoint was OS and secondary endpoints included type and duration of subsequent therapy.

**Results:**

Patients were a median age of 62.5 years; 48% had visceral metastases. At a median follow-up of 89.7 months, the median OS was 85.4 months (95% CI 64.3–not estimable). Median OS was longer in patients with nonvisceral versus visceral metastases (not reached vs 67.3 months), or with treatment-free interval > 12 months versus ≤ 12 months (85.4 vs 45.4 months), or with treatment duration ≥ 24 months versus < 24 months (not reached vs 47.5 months). Of patients who received a first subsequent therapy (81%), most (67%) continued endocrine-based therapy, while 7% received chemotherapy. The median duration of the first subsequent therapy was 8.3 months (95% CI 3.9–12.2), and the median chemotherapy-free survival was 69.1 months (95% CI 24.2–85.4).

**Conclusions:**

In this population of Japanese women with ER+/HER2− ABC, median OS was over 7 years with 1L PAL + LET, supporting the use of 1L PAL + endocrine therapy.

**Trial number:**

NCT04735367.

**Supplementary Information:**

The online version contains supplementary material available at 10.1007/s12282-023-01511-z.

## Introduction

In Japan, breast cancer is the most common cancer in women, with an estimated 94,300 new cases diagnosed in 2022, and is now the fourth leading cause of cancer-related death in women [1, 2]. Breast cancer incidence in Japanese women has risen in recent decades [3], with a net drift of 1.78% (95% confidence interval [CI] 1.30–2.26%) from 1990 to 2019 and is projected to remain at historically elevated levels over the next decade [4]. Japanese women with breast cancer that has metastasized beyond the breast and proximal lymph nodes have a 5-year survival rate of 39.3% [5], highlighting the need for effective therapies for this patient population.

For postmenopausal women with estrogen receptor-positive/human epidermal growth factor receptor 2-negative (ER+/HER2−) advanced breast cancer (ABC), a cyclin-dependent kinase (CDK4/6) inhibitor combined with an aromatase inhibitor (AI) is strongly recommended as first-line therapy by the Japanese Breast Cancer Society [6]. Palbociclib, an orally active, selective CDK4/6 inhibitor, when combined with endocrine therapy (ET) has demonstrated efficacy and safety in the PALOMA-1 and 2 clinical trials for the treatment of women with ER+/HER2− ABC [7, 8]. Patients enrolled in PALOMA-1 were primarily from Western nations and demonstrated prolonged progression-free survival (PFS) for palbociclib plus letrozole compared with letrozole alone (20.2 vs 10.2 months; hazard ratio [HR] 0.488; 95% CI 0.319–0.748; *P* = 0.0004) [7] and numerically prolonged overall survival (OS) (37.5 vs 34.5 months; stratified HR 0.897; 95% CI 0.623–1.294; *P* = 0.281) [9]. PALOMA-2 enrolled a more diverse patient population [8] and demonstrated a PFS benefit for palbociclib plus letrozole versus placebo plus letrozole (27.6 vs 14.5 months; HR 0.563; 95% CI 0.461–0.687; *P* < 0.0001) [10] and numerically prolonged OS (53.9 vs 51.2 months; HR 0.956; 95% CI 0.777–1.177; *P* = 0.3378) [11]. A prespecified exploratory subgroup analysis of Japanese patients enrolled in PALOMA-2 revealed a numerical PFS advantage for palbociclib plus letrozole (n = 32) over placebo plus letrozole (n = 14) of 22.2 months versus 13.8 months (HR 0.59; 95% CI 0.26–1.34; *P* = 0.1027) [12]. On the strength of the PFS findings, palbociclib was approved for the treatment of patients with ABC in Japan in September 2017 [13].

To date, most breast cancer research has focused on Western patient populations [14]. To address gaps in knowledge regarding the efficacy of palbociclib plus letrozole for Japanese patients with ER+/HER2− ABC, an open-label, single-arm, phase 2 study was conducted in Japan (J-Ph2) [15]. Initial results showed a 1-year PFS probability of 75.6% (90% CI 62.4–84.7%) and an objective response rate of 40.5% (95% CI 25.6–56.7%) [15]. A follow-up of the J-Ph2 reported a median PFS of 35.7 months (95% CI 21.7–46.7), a manageable treatment safety profile, and no clinically meaningful deterioration in quality of life [16]. Here, we report the interim results of a planned extended follow-up of J-Ph2, evaluating OS and subsequent therapy use, in women with ER+/HER2− ABC.

## Patients and methods

### Study design and patients

This study (NCT04735367) is a planned follow-up of a phase 2, single-arm, open-label, multicenter study in Japan (J-Ph2, NCT01684215) [15, 16]. Patients were postmenopausal women with ER+/HER2− ABC who were treated with palbociclib plus letrozole in J-Ph2 and followed up for survival. This study had no exclusion criteria. Detailed inclusion and exclusion criteria for J-Ph2 have been published previously [15, 16].

This follow-up study was approved by the Institutional Review Board of each participating center and was conducted according to applicable local laws and regulatory requirements, the Ethical Guidelines for Medical and Health Research Involving Human Subjects issued by the Minister of Health, Labour and Welfare (MHLW), and the Declaration of Helsinki. For all living participants, informed written consent for continued participation in this study was obtained; for participants who had passed away before this study, participants’ legal representatives were notified of the conduct of this study and given the opportunity to refuse data collection. OS and subsequent therapy data were collected from individual patient medical records. Patient demographics and other relevant data collected in the J-Ph2 study were used and matched for analysis with data collected in this study through patient IDs.

### Study treatment

Patients initially received oral palbociclib at a starting dose of 125 mg/day given with food for 21 days, followed by 7 days off per 28-day cycle. Patients also received oral letrozole, 2.5 mg/day, continuously. Treatment was managed according to protocol requirements during J-Ph2 (details published previously [15]) and was subsequently managed by clinicians according to palbociclib Japanese label guidelines following the conclusion of J-Ph2.

### Outcomes

The primary endpoint of the study was OS, defined as the time from the first dose of study treatment (palbociclib plus letrozole) in J-Ph2 to date of death due to any cause. Secondary endpoints included type and duration of subsequent therapies. Chemotherapy-free survival (CFS) assessment was planned as an additional analysis. CFS was defined as the time from first dose of study treatment in the J-Ph2 study until the start of first subsequent chemotherapy or death due to any cause, whichever came first.

### Statistical analyses

All efficacy analyses were performed using data from all enrolled patients who received ≥ 1 dose of study medication as of the data cutoff date. Patient demographics and disease characteristics were summarized with descriptive statistics. Median OS, duration of subsequent therapy, and CFS and associated 95% CIs were estimated using the Kaplan–Meier method. Median OS was also assessed for baseline demographic and disease characteristic subgroups: visceral or nonvisceral metastatic disease; bone-only metastases or other metastases; treatment-free interval (TFI) from completion of prior adjuvant therapy (patients with endocrine-resistant disease [≤ 12 months], patients with endocrine-sensitive disease [> 12 months], or de novo metastatic disease); age (< 65 years, ≥ 65 years); duration of study treatment (< 24 months, ≥ 24 months); dose reduction (yes or no); Eastern Cooperative Oncology Group performance status (ECOG PS; 0 or 1); prior therapy (prior or no prior hormonal therapy; prior or no prior chemotherapy); and Ki67 status (> 20% or ≤ 20%). Median CFS was also assessed by visceral or nonvisceral metastatic disease and TFI.

## Results

### Patient population

A total of 42 patients were enrolled in the J-Ph2 study. At the end of J-Ph2, 8 patients had died, 30 continued follow-up, and 4 refused further follow-up. Of the 30 patients that continued follow-up at the end of J-Ph2, 28 patients were enrolled in this follow-up study, and 2 patients refused further follow-up (Table S1). Patients in the original cohort had a median age of 62.5 years (Table 1). Most patients (92.9%) had an ECOG PS of 0, while 7.1% had a score of 1. About half of patients (47.6%) had visceral metastatic disease, and 14.3% had bone-only metastatic disease. Approximately half of patients (47.6%) had a TFI > 12 months, and 33.3% had de novo metastatic disease.

**Table 1:** Patient demographics and baseline disease characteristics [15, 16]

Demographic or disease characteristic	Palbociclib + letrozole (n = 42)
Age, median (range), years	62.5 (43–84)
Weight, median (range), kg	50.4 (38.6–74.5)
ECOG PS, n (%)	
0	39 (92.9)
1	3 (7.1)
Disease site, n (%)	
Visceral	20 (47.6)
Nonvisceral	22 (52.4)
Bone only	6 (14.3)
TFI, n (%)	
≤ 12 months	8 (19.0)
> 12 months	20 (47.6)
de novo metastatic	14 (33.3)
Prior (neo)adjuvant therapies, n (%)	
Hormone therapy	27 (64.3)
Chemotherapy	20 (47.6)
Ki67-positive expression, n (%)	
≤ 20%	19 (45.2)
> 20%	23 (54.8)

*ECOG PS* Eastern Cooperative Oncology Group performance status, *HER2* human epidermal growth factor receptor 2, *TFI* treatment-free interval

### Overall survival

**Fig. 1:**
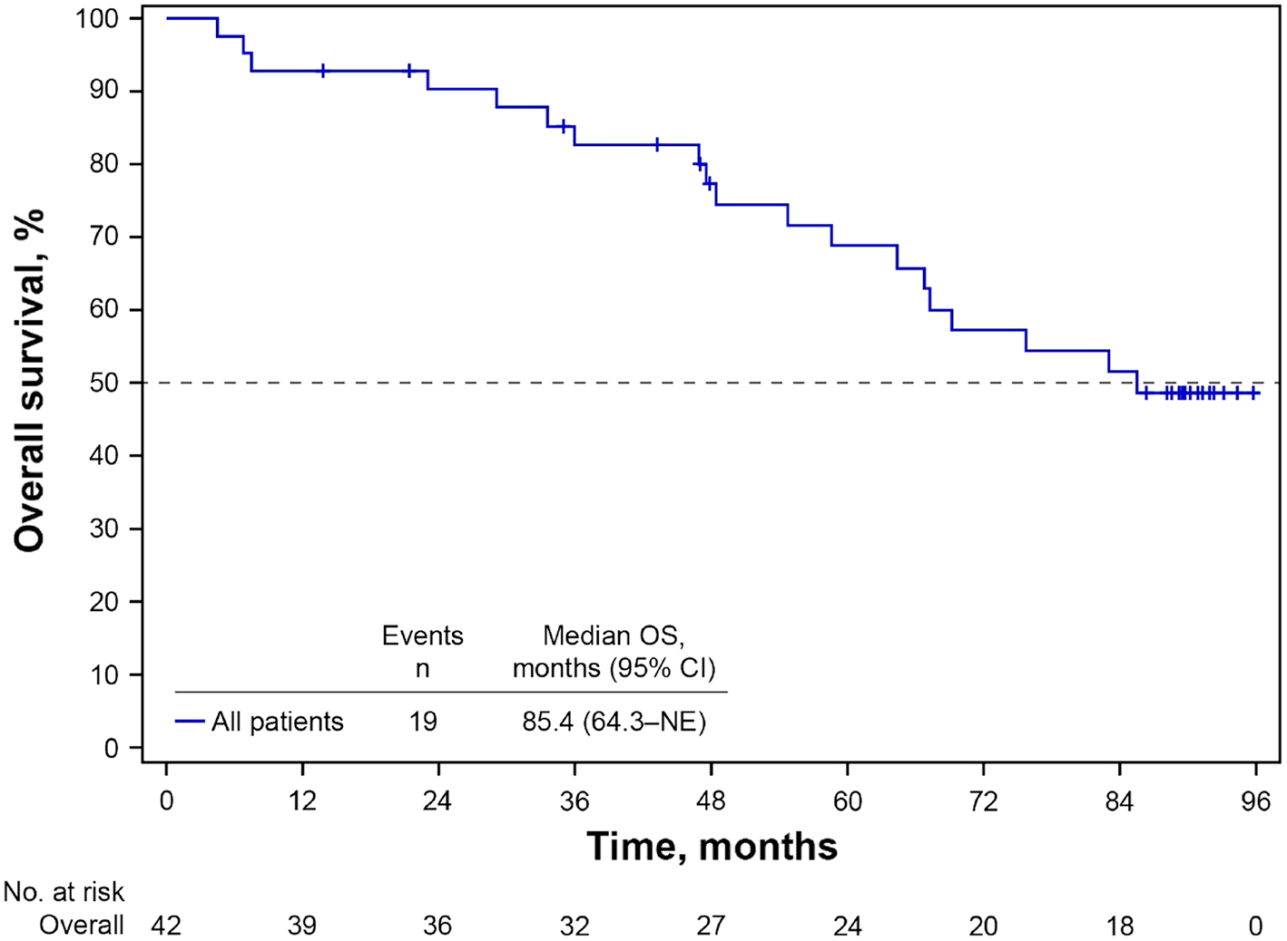
Kaplan–Meier estimated overall survival probability. *CI* confidence interval, *NE* not estimable, *OS* overall survival

**Fig. 2:**
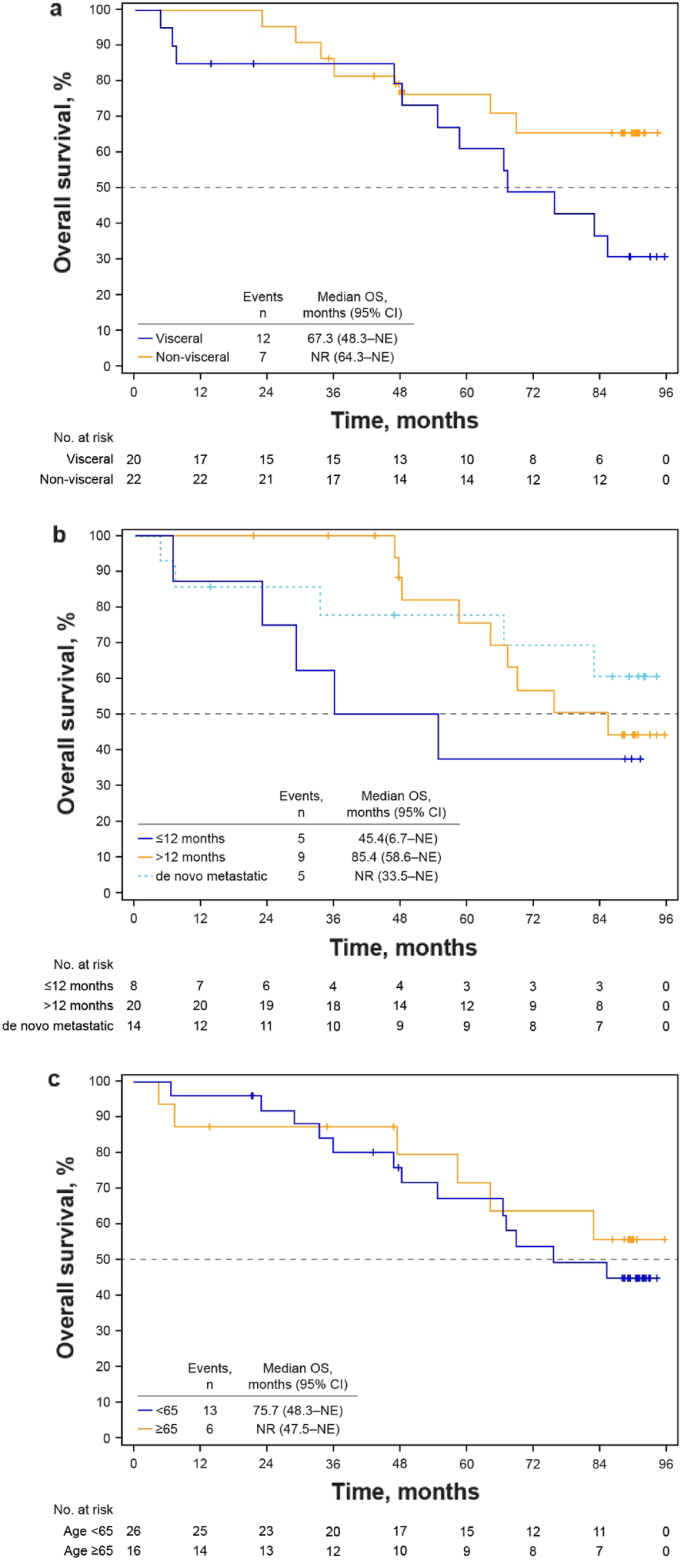
Kaplan–Meier estimated overall survival probability by **a** visceral versus nonvisceral metastases, **b** treatment-free interval (≤ 12 months vs > 12 months vs de novo metastatic), and **c** age (< 65 years vs ≥ 65 years). *CI* confidence interval, *NE* not estimable, *NR* not reached, *OS* overall survival

**Fig. 3:**
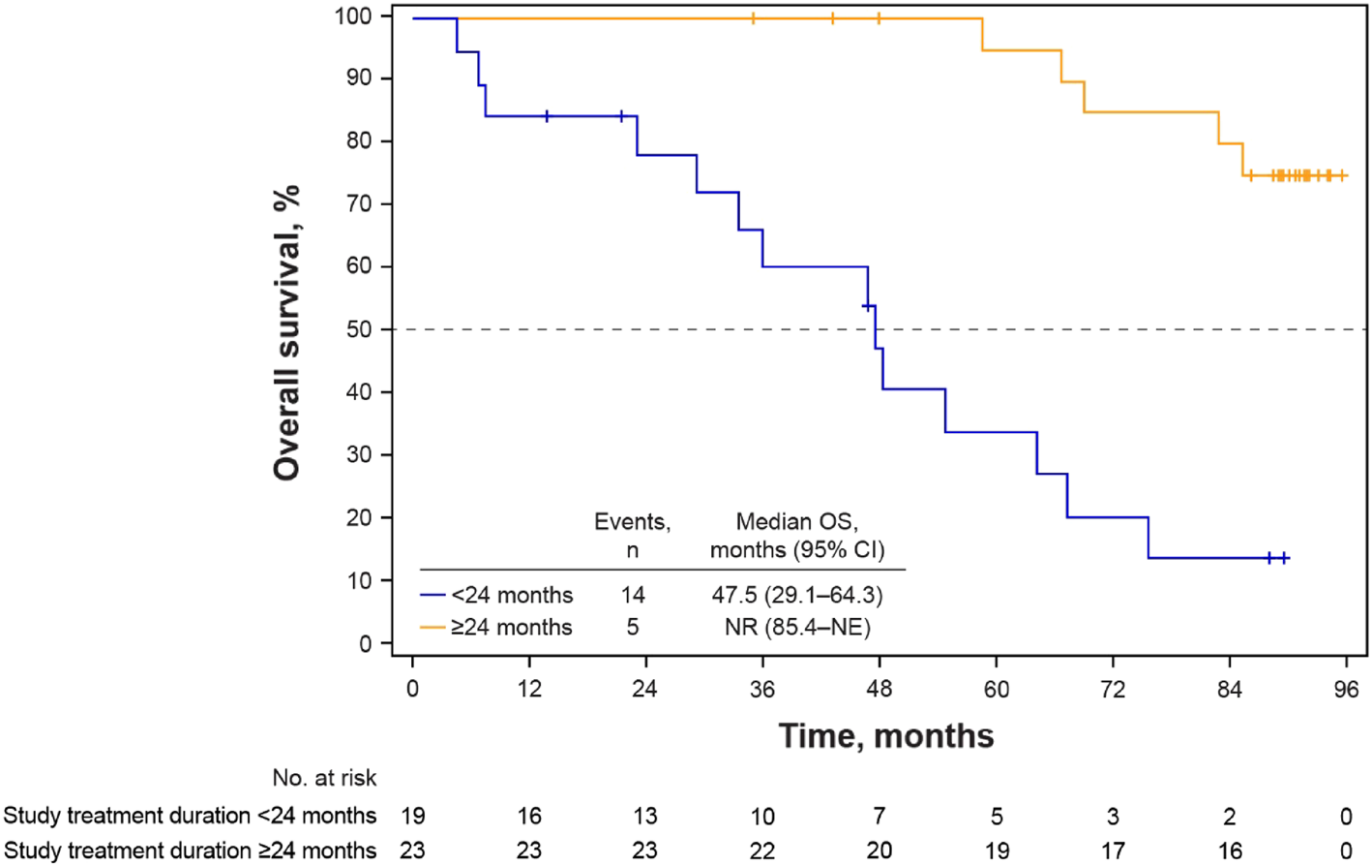
Kaplan–Meier estimated overall survival probability by duration of study treatment. *CI* confidence interval, *NE* not estimable, *NR* not reached, *OS* overall survival

At a median follow-up of 89.7 months, the median OS of patients during the study was 85.4 months (95% CI 64.3–not estimable [NE]) (Fig. 1). When analyzed by baseline characteristic subgroups, median OS was longer in patients without visceral disease than those with visceral disease (not reached [NR] vs 67.3 months), in those with TFI > 12 months, or de novo metastatic disease versus TFI ≤ 12 months (85.4 months or NR vs 45.4 months) and in those aged ≥ 65 years versus < 65 years (NR vs 75.7 months) (Fig. 2). When analyzed by duration of study treatment, patients who received palbociclib plus letrozole for ≥ 24 months had a longer median OS than those who received palbociclib plus letrozole for < 24 months (NR vs 47.5 months) (Fig. 3). Median OS was longer in patients with bone-only disease versus those with metastatic disease at other sites (NR vs 75.7 months) and in patients whose palbociclib dose had been reduced versus those with no dose reduction (NR vs 54.7 months) (Fig. S1). Other subgroup analyses are shown in Table S2.

### Dose and duration of study treatment

Treatment dose and duration of use for each patient are shown in Fig. S2. All patients initiated palbociclib at 125 mg/day. Of the 42 patients enrolled in J-Ph2, 10 (23.8%) did not have a palbociclib dose reduction, 19 (45.2%) had a single dose reduction from 125 to 100 mg/day, and 13 (31.0%) had their palbociclib reduced in 2 steps to 75 mg/day. At the end of this study, 3 patients (7.1%) were continuing to receive palbociclib plus letrozole.

### Subsequent therapy

**Fig. 4:**
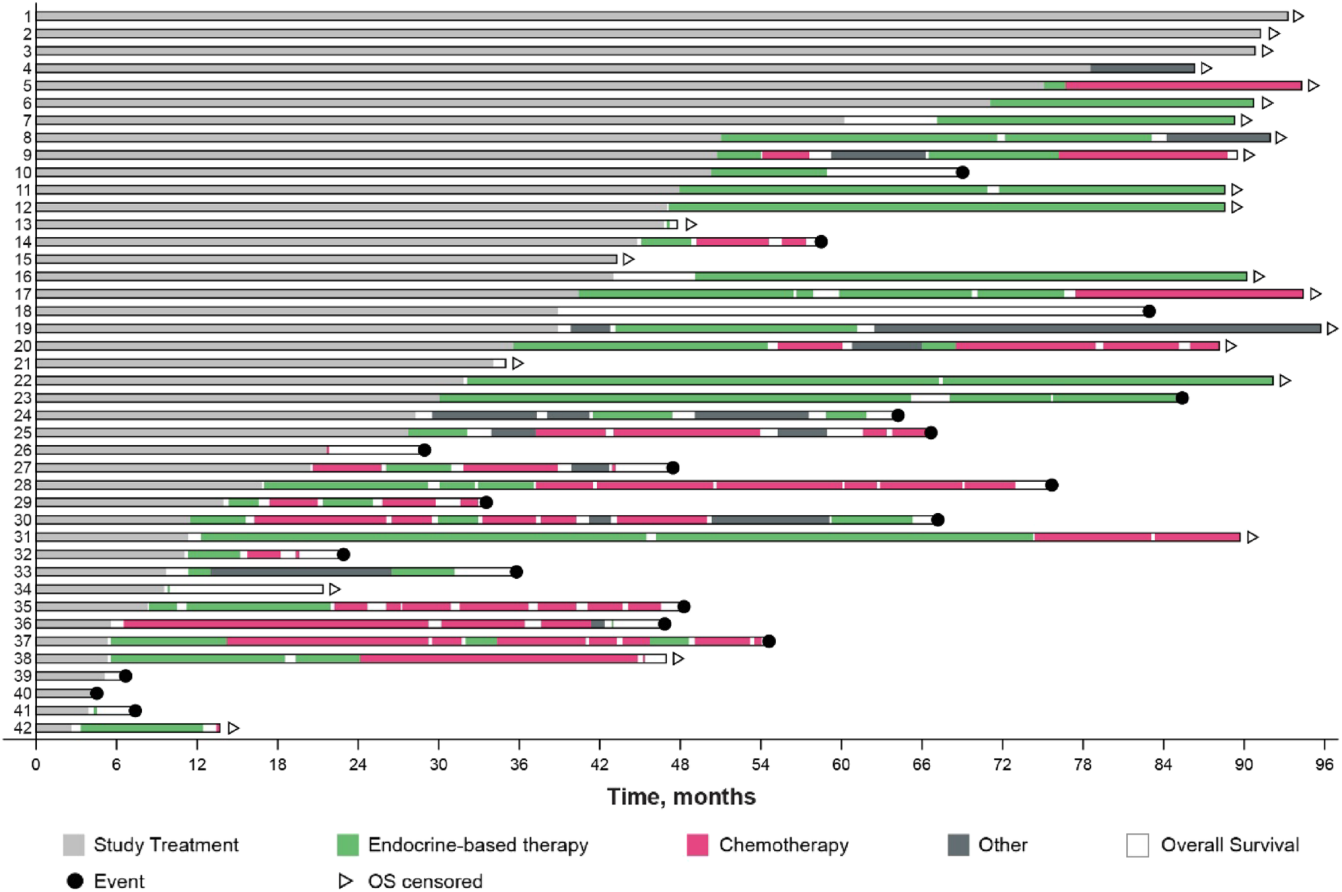
Type and duration of therapies. *OS* overall survival

Therapy patterns for each patient are shown in Fig. 4. Subsequent therapy was administered to 34 of 42 patients (81.0%, Table S3) for a median treatment duration of 8.3 months (95% CI 3.9–12.2) (Table 2). Of these patients, a majority (82.4%) received an endocrine-based therapy; 18 received ET alone including 12 who received fulvestrant. Seven patients received a CDK4/6 inhibitor plus ET, and 3 received everolimus plus ET. Three patients received chemotherapy as a first subsequent therapy, and 3 patients received other treatments. Of the 42 patients in the J-Ph2 study, 28 (66.7%, Table S3) received a second subsequent therapy with a median treatment duration of 5.8 months (95% CI 3.3–13.5) (Table 2). Sixteen of these 28 patients (57.1%) received another endocrine-based therapy as a second subsequent therapy; 10 received ET monotherapy including 5 who received fulvestrant, 3 received a CDK4/6 inhibitor plus fulvestrant, and 3 received everolimus plus ET. Nine patients received chemotherapy as a second subsequent therapy, and 3 received other treatments.

**Table 2:** Duration of subsequent therapy and chemotherapy-free survival

	N	Median time, months, (95% CI)
Duration of subsequent therapy		
First subsequent therapy	34	8.3 (3.9–12.2)
Second subsequent therapy	28	5.8 (3.3–13.5)
Chemotherapy-free survival		
Overall	42	69.1 (24.2–85.4)
Treatment-free interval		
≤ 12 months	8	18.9 (6.7–NE)
> 12 months	20	69.1 (37.4–NE)
de novo metastatic	14	65.5 (13.6–NE)
Disease site		
Visceral	20	37.3 (13.6–76.8)
Nonvisceral	22	77.5 (49.2–NE)

*CI* confidence interval, *NE* not estimable

**Fig. 5:**
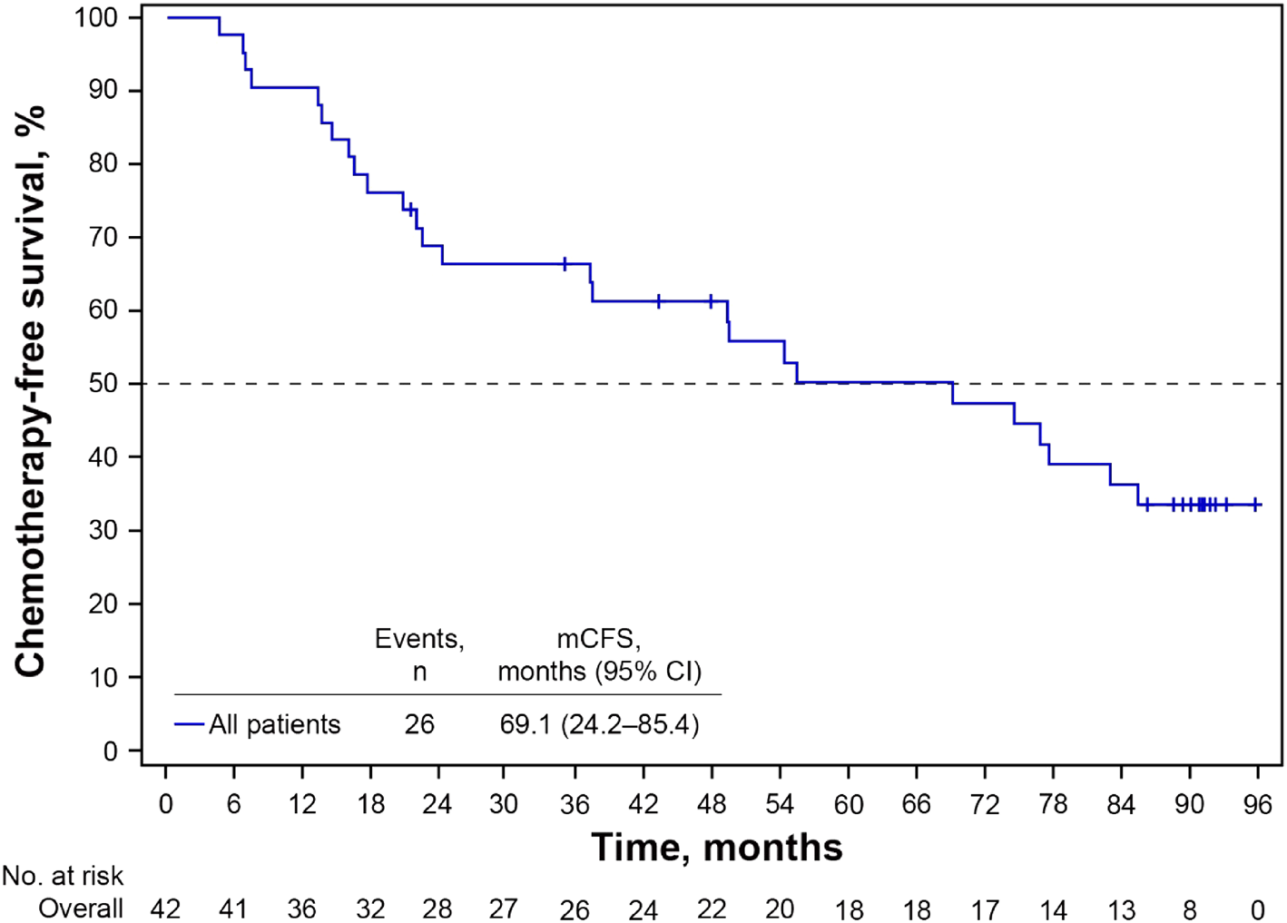
Kaplan–Meier estimated chemotherapy-free survival. *mCFS* median chemotherapy-free survival, *CI* confidence interval

Median CFS was 69.1 months (95% CI 24.2–85.4) (Fig. 5). When analyzed by TFI and disease site subgroups (Table 2), median CFS was longer for patients with TFI > 12 months, or de novo metastatic disease than for those with TFI ≤ 12 months (69.1 or 65.5 vs 18.9 months, respectively), and for patients with nonvisceral disease than for those with visceral disease (77.5 vs 37.3 months).

## Discussion

After a median follow-up of over 7 years, we report a remarkably long median OS of 85.4 months for Japanese patients with ER+/HER2− ABC who were treated with first-line palbociclib plus letrozole. A majority of patients in this study continued to receive ET in the second (82.4%) and third line (57.1%). Furthermore, the median CFS of 69.1 months was notably prolonged.

While interstudy comparisons must be made with caution, it is informative to view the current findings within the context of related studies. Mature OS data are still rare for Asian patients with ABC who have been treated with CDK4/6 inhibitors, limiting the number of studies that can provide context for the current results. The median OS reported here (85.4 months) compares favorably with the median OS (53.9 months) reported in the palbociclib plus letrozole arm of the PALOMA-2 trial [11]. Although the sample sizes of the 2 studies were different (42 in J-Ph2 vs 444 in PALOMA-2) and precise comparison is challenging, similar baseline patient demographics and disease characteristics were observed for patient median age (62.5 vs 62 years), percentage of patients with visceral disease (47.6% vs 48.2%), endocrine-resistant disease (TFI ≤ 12 months; 19.0% vs 22.3%), de novo metastatic disease (33.3% vs 37.6%), ≤ 20% Ki67-positive expression (45.2% vs 58%) and patients with previous (neo)adjuvant ET (64.3% vs 56.1%) [8, 10, 17]. In contrast, the studies differed markedly in the percentage of patients with an ECOG PS of 0 (92.9% vs 57.9%) [8, 15]. However, the median OS for the ECOG PS 0 subgroup in PALOMA-2 was 58.2 months [11], making it unlikely that the higher proportion of patients with ECOG PS 0 alone would account for the difference in median OS observed between the studies.

There were substantial differences between this study and PALOMA-2 in the racial and geographical characteristics of patients [8, 15]. The PALOMA-2 trial only included 13.8% of patients from the Asia-Pacific region, whereas those in the J-Ph2 study were all from Japan. Notably, the OS observed in the Asia-Pacific region subgroup analysis of PALOMA-2 (73.4 months) more closely mirrors our results [11]. Further, the OS HR for the Asia-Pacific region subgroup was lower than that of the overall population (0.74 vs 0.96, respectively), suggesting that region and/or race could be contributing factors in palbociclib plus ET efficacy [11]. OS data from the PATHWAY trial comparing palbociclib plus tamoxifen versus placebo plus tamoxifen in Asian patients with ABC, though not yet mature, also showed a trend toward prolonged OS in the palbociclib plus tamoxifen arm (HR 0.73; 95% CI 0.442–1.207) [18]. An OS benefit for a CDK4/6 inhibitor plus letrozole was also observed in the Asia geographic subgroup of the MONALEESA-2 trial, which reported a median OS of 65.3 months with ribociclib plus letrozole versus 51.2 months with placebo plus letrozole [19]. However, an OS benefit was not found for the Asian race subgroup; the median OS was only 51.0 months with ribociclib plus letrozole compared with 52.5 months with placebo plus letrozole [19]. Given the modest numbers of patients in these subgroups and in J-Ph2, defining the role of cultural, environmental, or genetic factors in determining OS for patients treated with palbociclib plus ET will require further investigation.

The PALOMA-3 trial identified four significant prognostic factors of OS benefit with palbociclib treatment including nonvisceral disease, ECOG PS 0, endocrine sensitivity, and no prior chemotherapy for ABC [20]. Our subgroup analyses also showed considerably longer median OS in patients with nonvisceral disease, no prior chemotherapy, and TFI ≥ 12 months, but as observed in PALOMA-3 [20], age did not have an impact on OS benefit. In our analysis, patients with palbociclib plus letrozole treatment duration ≥ 24 months had longer median OS than those with treatment duration < 24 months, further supporting the association of palbociclib plus letrozole use and OS benefit. A longer duration of exposure to the study treatment may have been achieved in part through dose management as palbociclib treatment duration has been shown to correlate with dose reductions [21]. The relationships between patient OS and palbociclib treatment patterns and duration warrant further investigation.

The median CFS reported here (69.1 months) and the median PFS previously reported for this study (35.7 months) [16] also compare favorably with those reported in the palbociclib plus letrozole arm of PALOMA-2 (38.1 months and 27.6 months, respectively) [10, 11]. Of note, differences between the studies in CFS (delta of 31.0 months) and OS (delta of 31.5 months) were dramatically greater than the difference in PFS (delta of 8.1 months) [10, 11]. A potential reason for the extended CFS in this study may be the effectiveness of subsequent ET in second and third lines of treatment. Notably, a higher percentage of patients received second- and third-line ET in our study (82.4% and 57.1%) than in PALOMA-2 (60.8% and 36.2%) [10]. The treatment duration of these second- and later-line ETs sometimes exceeded the duration of previous treatments, potentially indicating that treatments with higher response rates had been identified and chemotherapy could be postponed. Understanding the factors underlying the delay in time to chemotherapy is particularly important since chemotherapy negatively impacts patients’ quality of life [22].

Various components of the cyclin D:CDK4/6:retinoblastoma pathway have been assessed as potential mechanisms of resistance to CDK4/6 inhibitor plus ET therapy [17]. Biomarker analyses from PALOMA-2 have shown that higher *ESR1* expression levels, an indicator of estrogen sensitivity, were associated with a PFS benefit for patients taking either palbociclib plus letrozole or placebo plus letrozole [17]. The same study reported that higher levels of CDK4 were associated with reduced placebo plus letrozole efficacy (but not palbociclib plus letrozole efficacy), indicating a potential link between CDK4 expression and endocrine resistance; levels of cyclin D/E, CDK6, and retinoblastoma were not correlated with palbociclib plus letrozole efficacy [17]. Furthermore, evidence from the PALOMA-2/3 trials indicates that palbociclib may restore estrogen-sensitivity in previously estrogen-resistant tumors [23]. Studies that have evaluated novel endocrine monotherapies on patients that had progressed on CDK4/6 inhibitors plus ET have yielded mixed results, with a promising PFS benefit for elacestrant [24] but not for venetoclax [25], highlighting the heterogeneity in ET efficacy. Most patients in our study received second-line ET treatment with a median treatment duration of approximately 8 months, suggesting that appropriate therapies had been identified by the treating physician. Although subsequent therapies were not selected based on resistance mechanisms in our study, it is possible that these treatment decisions contributed to the extended CFS and prolonged OS.

Real-world data (RWD) studies are necessary to evaluate the effectiveness of treatments in routine clinical practice, and often include patients who are older, more heterogeneous in ethnic and racial background, and frequently do not meet the strict requirements for inclusion in clinical trials [26, 27]. In contrast with the PALOMA trials, a real-world analysis using data from the Flatiron Database found a significantly prolonged median OS for patients with HR+/HER2− metastatic breast cancer being treated with palbociclib plus an AI versus an AI alone in routine clinical practice (49.1 vs 43.2 months; HR 0.76; 95% CI 0.65–0.87; *P* < 0.0001) [28]. Another study using RWD from the Surveillance, Epidemiology and End Results (SEER)-Medicare database reported a 41% lower mortality rate with CDK4/6 inhibitors (90% used palbociclib) plus ET than with ET alone (multivariate-adjusted HR 0.59; 95% CI 0.423–0.823) in women aged ≥ 65 years with HR+/HER2− metastatic breast cancer [29, 30]. Though both reports are consistent with our study in showing an OS benefit associated with palbociclib plus ET, most patients enrolled were White. While multiple RWD studies have evaluated the effectiveness of palbociclib plus ET for patients in Asia, the OS data reported were not mature [31, 32]. This ongoing gap in knowledge highlights the importance of this study as well as the need for additional OS data for patients with ER+/HER2− ABC in the Asia-Pacific region.

Although palbociclib was the first-in-class CDK4/6 inhibitor approved for the treatment of patients with ABC, other CDK4/6 inhibitors, including abemaciclib and ribociclib, have also been approved as ABC therapies [33, 34]. The MONARCH 3 trial, evaluating the efficacy of abemaciclib plus nonsteroidal AI, enrolled 31.4% Asian patients [35] and reported a median OS of 67.1 months (interim results) [36]. The MONALEESA-2 trial, evaluating the efficacy of ribociclib plus letrozole, enrolled 8.4% Asian patients [37] and reported a median OS of 63.9 months (95% CI 52.4–71.0) [19], though ribociclib is not a currently approved therapy for ABC in Japan. These median OS results, while numerically longer than those reported in PALOMA-2, are still shorter than our results. Though data directly comparing CDK4/6 inhibitor efficacy are not yet available, the evidence to date indicates that CDK4/6 inhibitors may be effective in prolonging OS in Asian patients.

Some limitations of this study include the fact that it was a single-arm, open-label design and therefore did not have a placebo plus letrozole comparator arm. It also had a small sample size and may not be representative of the larger Japanese patient population. However, it had a remarkably extended median follow-up, enabling the description of a median OS exceeding 7 years and a median CFS of more than 5.5 years. The data presented here were collected in part during J-Ph2 in a clinical trial setting, where patient care was carefully managed by experienced investigators and site coordinators. As such, the current study differs somewhat from studies that take place entirely in the real-world setting. Investigator familiarity with the management of palbociclib may have contributed to the exceptional outcomes observed in this study.

## Conclusion

This initial interim analysis showed a median OS of over 7 years with first-line palbociclib plus letrozole, adding to the growing body of evidence supporting first-line palbociclib plus ET for the treatment of Japanese patients with ER+/HER2− ABC. This report provides insight into real-world subsequent treatment patterns following palbociclib plus letrozole.

### Supplementary Information

Below is the link to the electronic supplementary material.

Supplementary file1 (DOCX 652 kb)

## Data Availability

Upon request, and subject to review, Pfizer will provide the data that support the findings of this study. Subject to certain criteria, conditions and exceptions, Pfizer may also provide access to the related individual de-identified participant data. See https://www.pfizer.com/science/clinical-trials/trial-data-and-results for more information.
