# Supplementary material for: Overall survival in Japanese patients with ER+/HER2− advanced breast cancer treated with first-line palbociclib plus letrozole
Source: Breast Cancer. 2023 Oct 26;31(1):53–62. doi: 10.1007/s12282-023-01511-z (PMC10764519; doi:10.1007/s12282-023-01511-z)

# Supplementary Materials

### Table S1 Study disposition

| **Disposition** | **Palbociclib + Letrozole**  **n (%)** |
| --- | --- |
| Status at the end of J-Ph2 (NCT01684215)  Treated participants  Continued follow-up  Died  Refused further follow-up | 42  30 (71.4)  8 (19.0)  4 (9.5**)** |
| Status at the start of study NCT04735367  Enrolled for further follow-up  Refused further follow-up | 28 (93.3)^a^  2 (6.7)^a^ |
| Status at the data cutoff of study NCT04735367  Alive  Died | 17 (60.7)^b^  11 (39.3)^b^ |

^a^Calculated as a percentage of patients who continued follow-up at the end of J-Ph2. ^b^Calculated as a percentage of patients who enrolled in study NCT04735367 and agreed to further follow-up.

### Table S2 Median overall survival by subgroup

| **Subgroup** | | **N** | **Median OS  months (95% CI)** |
| --- | --- | --- | --- |
| Disease site | Visceral  Nonvisceral | 20  22 | 67.3 (48.3–NE)  NR (64.3-NE) |
|  | Bone-only  Other | 6  36 | NR (23.0–NE)  75.7 (58.6–NE) |
| TFI since completion of prior therapy | ≤12 months  >12 months  de novo metastatic | 8  20  14 | 45.4 (6.7–NE)  85.4 (58.6–NE)  NR (33.5–NE) |
| Age | <65 years  ≥65 years | 26  16 | 75.7 (48.3–NE)  NR (47.5–NE) |
| Dose reduction group | No  Yes | 10  32 | 54.7 (6.7–75.7)  NR (67.3–NE) |
| Duration of palbociclib treatment | <24 months ≥24 months | 19  23 | 47.5 (29.1–64.3)  NR (85.4–NE) |
| ECOG PS | 0  1 | 39  3 | 85.4 (64.3–NE)  NR (48.3-NE) |
| Prior therapy | Prior hormonal therapy  No prior hormonal therapy | 27  15 | 75.7 (54.7–NE)  NR (33.5–NE) |
|  | Prior chemotherapy  No prior chemotherapy | 20  22 | 75.7 (46.9–NE)  NR (66.7–NE) |
| Ki67 status | >20%  ≤20% | 23  19 | 67.3 (48.3–NE)  NR (64.3–NE) |

CI, confidence interval; ECOG PS, Eastern Cooperative Oncology Group Performance Status; HER2, human epidermal growth factor receptor 2; NE, not estimable; NR, not reached; OS, overall survival; TFI, treatment-free interval

### **Table S3** Type of subsequent therapy

|  | **Palbociclib + Letrozole**  **(N = 42)**  **n (%)** |
| --- | --- |
| No subsequent therapy received  Study treatment ongoing  Study treatment terminated | 8 (19.0)  3 (7.1)  5 (11.9) |
| Received first subsequent therapy^a^  Endocrine-based therapy  ET alone  Fulvestrant  CDK4/6 inhibitor + ET  CDK4/6 inhibitor + AI  CDK4/6 inhibitor + fulvestrant  Everolimus + ET  Chemotherapy  Bevacizumab + paclitaxel  Other  Investigation drug (+ ET/chemotherapy)  Other | 34 (81.0)  28 (82.4)  18 (52.9)  12 (35.3)  7 (20.6)  4 (11.8)  3 (8.8)  3 (8.8)  3 (8.8)  3 (8.8)  3 (8.8)  2 (5.9)  1 (2.9) |
| Received second subsequent therapy^b^  Endocrine-based therapy  ET alone  Fulvestrant  CDK4/6 inhibitor + fulvestrant  Everolimus + ET  Chemotherapy  Other  Investigational drug (+ ET/chemotherapy)  ET + chemotherapy | 28 (66.7)  16 (57.1)  10 (35.7)  5 (17.9)  3 (10.7)  3 (10.7)  9 (32.1)  3 (10.7)  2 (7.1)  1 (3.6) |

^a^Therapy percentages below are calculated based on number of patients receiving first subsequent therapy. ^b^Therapy percentages below are calculated based on number of patients receiving second subsequent therapy. AI, aromatase inhibitor; CDK4/6, cyclin-dependent kinase 4/6; ET, endocrine therapy

Fig. S1 Kaplan–Meier estimated overall survival probability by (a) bone-only disease versus other disease site; (b) patients who received dose reductions versus those who did not. CI, confidence interval

**a**


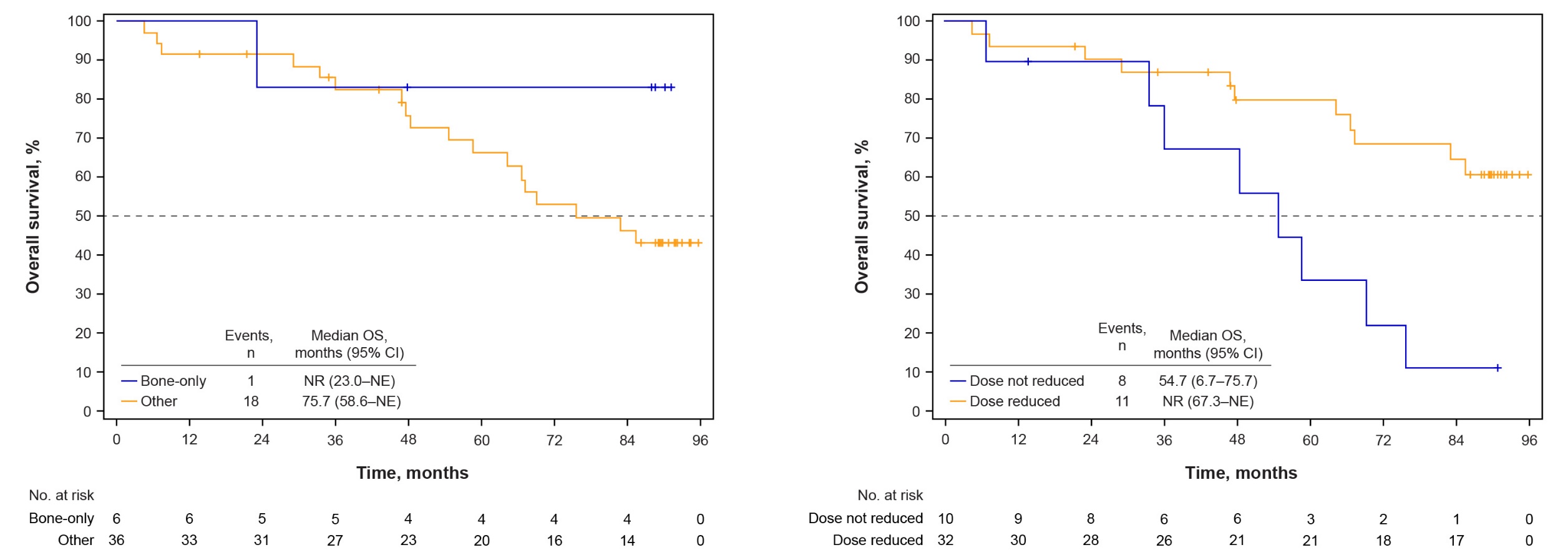


**b**


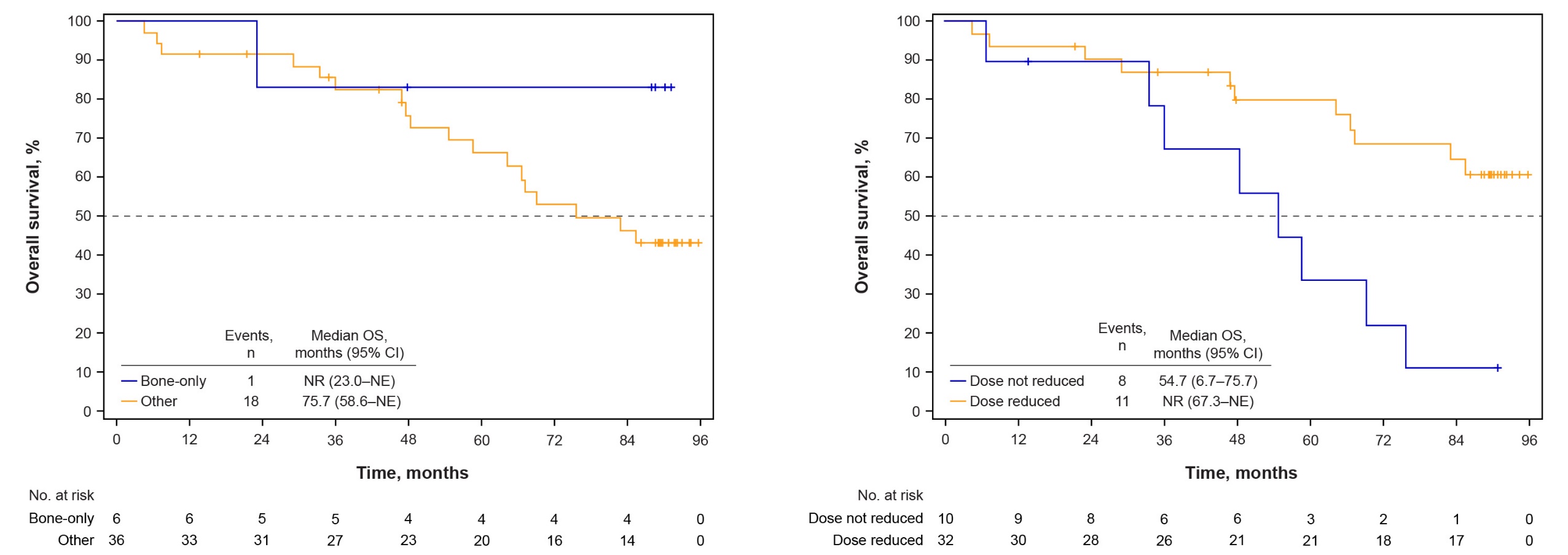


Fig. S2 Dose and duration of palbociclib treatment (Swimmer plot). LET, letrozole; PAL, palbociclib


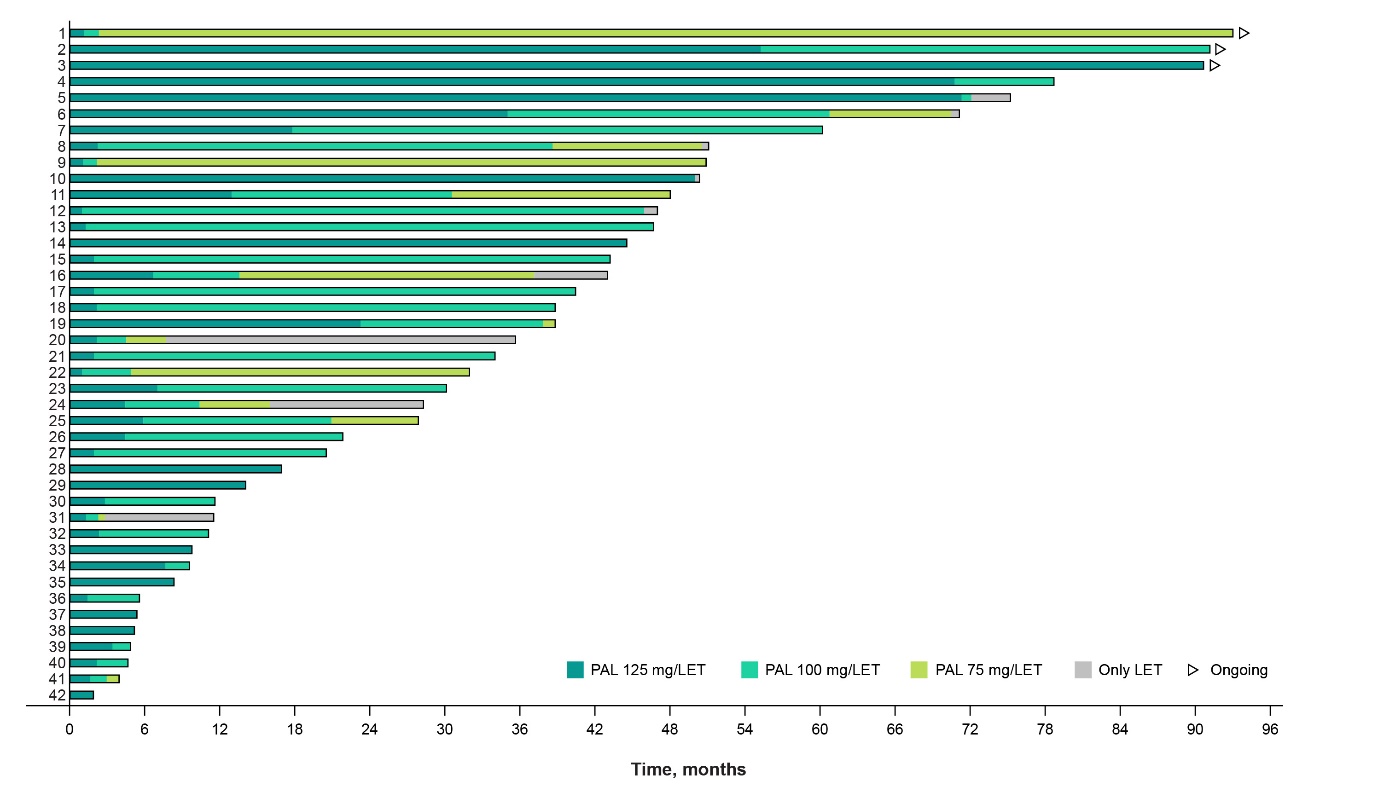

Supplement: Supplementary file 1 — Supplementary file1 (DOCX 652 kb) [file 12282_2023_1511_MOESM1_ESM.docx]
